# The integration of artificial intelligence with social Media: opportunities, challenges, and pathways for resource optimization and doctor-patient relationship enhancement in healthcare

**DOI:** 10.3389/fdgth.2026.1738784

**Published:** 2026-02-23

**Authors:** Hui Sun, Xiaowei Chen, Yi Yuan

**Affiliations:** Yantaishan Hospital, Yantai, Shandong, China

**Keywords:** artificial intelligence, digital health governance, doctor-patient relationship, health misinformation, social media

## Abstract

The integration of social media platforms and artificial intelligence (AI) has transformed the patient's role from that of a passive recipient to an active participant in healthcare navigation. The advent of short video platforms (such as TikTok and YouTube Shorts) has eliminated many conventional limitations related to location and time in medical education. Recent research suggests that sophisticated AI models (e.g., GPT-4) might outperform physicians in specific measurable aspects, such as diagnostic accuracy in controlled settings or empathy demonstrated through written communication. Nevertheless, physicians continue to be essential for coordinating complex care, resolving intricate ethical dilemmas, and maintaining the integrity of the physician-patient relationship. Consequently, although human participation remains essential, the digital environment is affected by integrity concerns. It is estimated that approximately 37% of medical social media posts contain misinformation, although this rate varies considerably among different health categories. To effectively resolve these challenges, we advocate for a collaborative stakeholder approach to governance. Through the implementation of formal platform certification, ongoing education for healthcare professionals, and AI-enabled filtering of user-generated content, we can improve the efficiency of medical resource allocations such as minimizing unnecessary inquiries—while laying a solid foundation for a sustainable, trust-based relationship between physicians and patients.

## Introduction

**Figure 1:**
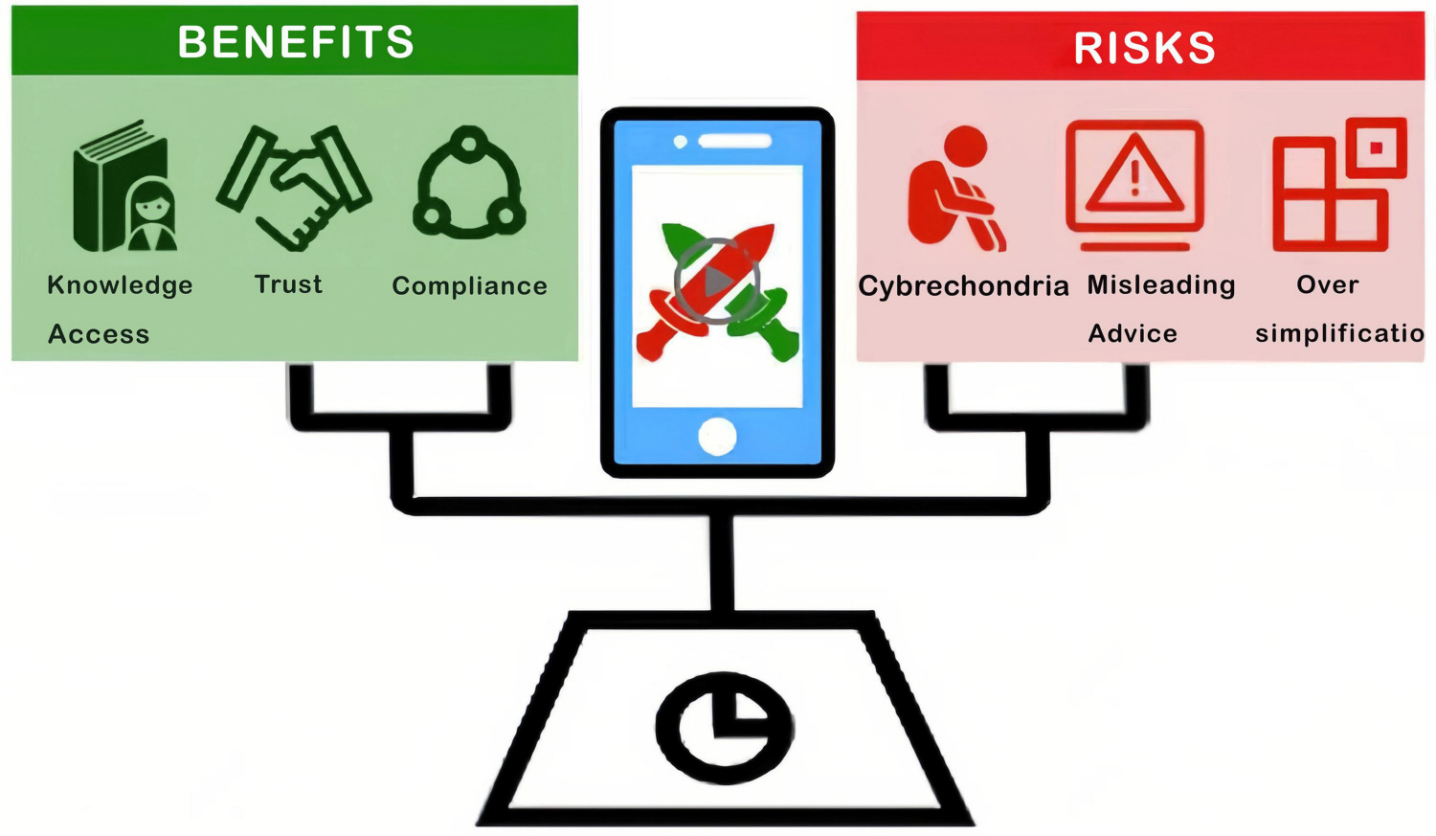
Double-Edged sword of medical short-videos.

YouTube and TikTok are two prominent examples of social media platforms with the largest user bases worldwide and the most accessible channels for disseminating health information. The primary strengths of these platforms reside in the user-friendly nature of their information formats—such as short videos and 3D animations—and their broad accessibility, reaching nearly 4.62 billion users globally. However, the quality of information varies considerably due to disparities in the professional backgrounds of content creators and differing content review standards, which means they do not constitute the “most reliable or clinically accurate” sources of health information (1). However, these platforms are a double-edged sword (Figure 1) since they make it easy for anyone to get information, but they also provide a lot of inaccurate or misleading content. These platforms are important sources of information, but the quality of the content might vary a lot. Social media connects healthcare providers, patients, and the public, thereby fundamentally changing the way in which patients seek and receive Health Information. Douyin (in China) has experienced over 50 billion views per year of its health-related content, demonstrating the extent to which the Public has transitioned to Visual forms of accessing Health Information. This study introduces an innovative method for bridging gaps in Governance by integrating Artificial Intelligence (AI) with Social Media Platforms. In this context, “integration” refers to the synergistic incorporation of AI algorithms (such as natural language processing and recommendation systems) into social media ecosystems to enhance content delivery and user engagement.

### I. The Main Value and Benefits of Short Videos for the Healthcare Industry

**1. Making it easier for people to learn and lessening the burden on doctors for basic consultations**

The ease of use of social media has made it easier for patients to find medical information and for the public to learn more about how diseases work (2). This transmission paradigm enables patients to access knowledge that was previously confined to professional domains, But the level of patient knowledge may not match the inherent difficulty of medical science. However, short videos use dual-channel processing (visual and aural) to greatly lower cognitive burden compared to standard text-based pamphlets. This is backed by Mayer's Cognitive Theory of Multimedia Learning (3). Nonetheless, it is essential to recognize the limitations of this theoretical applicability within health-related contexts. The efficacy of multimedia learning is significantly mediated by the user's eHealth literacy—defined as the ability to seek, discover, understand, and appraise health information from electronic sources. Individuals with limited digital literacy may continue to find it challenging to synthesize complex clinical topics despite the use of visual aides, requiring content strategies to be tailored to different proficiency levels as delineated in the eHealth Literacy Framework (4). Also, a meta-analysis from 2024 shows that video-based methods are better for learning in health education. This format makes complicated medical ideas (such surgical procedures) easier to understand by turning them into simple visual stories (5). This helps people who aren't doctors understand health issues and how to deal with them, even though thorough clinical expertise is still the job of professionals (6). Recent empirical research from Nature Human Behaviour suggests that social media usage, especially when interacting with credible sources, may positively correlate with enhanced belief correctness, indicating a possibility for authentic comprehension beyond mere access (7).

For instance, while talking about surgical diseases, the main people that supply information are healthcare professionals, such as surgeons and anesthesiologists who work at Grade A tertiary hospitals. Before dividing the operation down into three parts, preoperative preparation, intraoperative procedures, and postoperative recovery—content providers teach their audience about the reasons for surgery and how it might be used. For example, preoperative preparation: emphasis is placed on describing the examination items, assessing risk, and discussing the critical elements of preoperative preparations for patients. Within the intraoperative procedures, two distinct formats exist. The first format is the use of 3D animations to replicate the surgical incision site, the technique of tissue dissection, and the method of removal of the lesion, while avoiding graphic representation of the procedure and clearly indicating the operational logic behind the animation. In the second style, a doctor uses models and diagrams to explain the basic ideas behind surgery (for example, “pipe unclogging” for vascular interventional surgery and “repair of a wall” for hernia surgeries. In terms of postoperative recovery, it is focused on the major points of care, prevention of complications, and timelines for recovery. Most audience concerns regarding how long it will take to return to their normal life, and what precautions need to be taken after the surgery, are addressed in the recovery section. The recovery segment answers most of the audience's questions about how long it will take to get back to normal and what precautions they need to take after the surgery. Sharing complicated medical information can help patients stick to their treatment plans (which will be talked about more in this paper), but it also has risks. For example, general recovery timelines may not accurately show how different each patient is (for example, patients with other medical conditions), which could make patients who are “outliers” or who are misjudging how fast their recovery is going feel anxious for no reason.

The dissemination model has created greater public awareness about disease treatment and health management. In fact, surveys indicate that approximately 70% of surgeons surveyed in the United States and 52.7% of urologists in China believe that social media can play an important role in medical education. Social media also acts as a supportive environment where users can find psychological support and receive information about treatments through peer-to-peer relationships. For women, in particular, social media has provided a means to find others who have similar health concerns, discuss their treatment options with other women, and receive support from fellow users without feeling uncomfortable about being private and obtaining professional medical advice later (8,9). Furthermore, the dissemination model gives patients a reasonable expectation of how long a specific medical process may take, which reduces anxiety and miscommunication regarding the time required to complete a medical process due to asymmetrical information and lays the groundwork for creating trust between patients and physicians.

Douyin is a great example, as the application continues to grow rapidly. Between January 2024 and January 2025, Douyin added more than four million medical science popularization videos for users to view (with more than 50 billion views in total) (2,10). This has significantly decreased the volume of “ routine inquiries” for physicians and allowed for optimal time usage when attending important clinical areas of expertise. Furthermore, because of Douyin's increasing popularity, patients can anonymously ask questions regarding private medical issues. This meets the need for patients to feel comfortable receiving help from healthcare professionals and allows patients to network during their healing processes. Lastly, Douyin's growth also cuts down on unnecessary referrals to outpatient treatments, which makes it easier for primary care physicians to handle referrals (11).

**2. Getting professional resources to work and spreading the word about the value of doctors**

**Figure 2:**
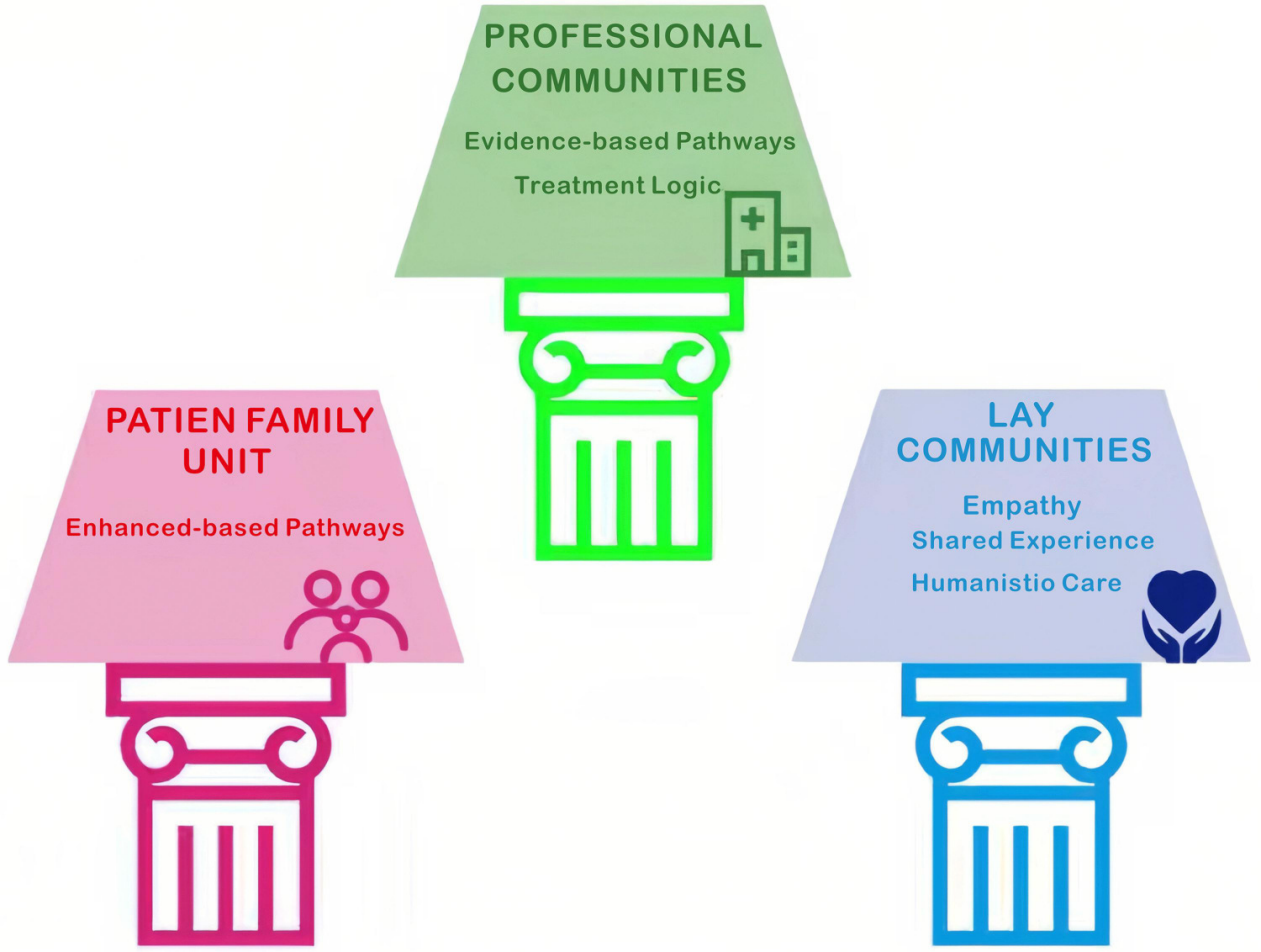
Professional-eMOTIONAL dUAL support framework.

Social media has developed into an essential method for medical and professional educators to have physician-created brands, educate patients and provide physician collaboration. In addition to being a broadcasting channel, social media has transformed into an interactive environment for the professional development of physicians and other healthcare workers through the gradual elimination of traditional hierarchy by giving physician creators and experienced researchers immediate access to large public audiences. Social media encourages healthcare professionals to work together and share information, training, and resources across distances and time zones (9,12) As social media has continued to mature, its impact on educated and highly skilled health professionals, communities, and patients continues to grow exponentially. Evidence of this growing impact can be seen in 2024–2025 on the Douyin platform where the number of individually certified medical content creators increased from 18,000 to 71,000 within a two-year period (over 50% of whom are employed at Grade A tertiary hospitals) and the number of institutionally certified medical content creators is over 5,000. Social media has made it easier for healthcare professionals to talk to each other and share medical resources, breaking down geographic and hierarchical boundaries in the healthcare community. Many studies support the assertion that plastic surgeons with a significant following on social media can better communicate with and educate their patients through social media platforms than small practices (10). This is especially beneficial when patients can review a physician's experience and accomplishments prior to making an appointment, thus enhancing the process of developing trust. Social media is also useful for patients with specific conditions or worries that need personalized care, such as “pediatric” diseases and “oncology” (13,14). It also lets families of patients with chronic illnesses connect with each other as “online support communities.” The “island effect” of being alone for families dealing with chronic illnesses causes them to feel very stressed and burdened. In response to the difficult burden of the “island effect,” we propose the “dual support framework” illustrated in Figure 2. The dual support framework consists of the professional community and the lay community supporting one another and working toward common goals. Professional communities, which provide the “hard evidence” of scientifically based treatment options, work to correct patients” cognitive biases, while lay communities offer the “soft power” of humanistic care through shared individual experiences that cannot be replicated by physicians. Figure 2 shows that the AI-driven ecosystem connects these two groups. This lets professional guidelines flow into lay discussions, which helps stop the spread of inaccurate or misleading content in patient support groups. Both pillars are essential to creating a healthy family unit for patients dealing with chronic illness.

**3. AI-Algorithm Collaboration Enhancing the Efficiency of Physician Resource Allocation**

Artificial intelligence (AI) and platform algorithms combine to create an improved way of delivering health information and utilizing medical resources. With platforms using collaborative filtering algorithms (using user preferences) and content feature matching (comparing topics), platforms can provide people with personalized recommendations of health-related information based upon their behavior. This means that people looking for specific health information, like how to manage diabetes or mental health problems, will get more accurate information that is directly relevant to them. This will cut down on the amount of irrelevant health information that is sent to users, which will make it easier for doctors to give them popular science content that meets their needs.

AI technology also gives doctors and nurses new tools that help them quickly look at a lot of health-related content on social media. For instance, sentiment-analysis technologies can find out how people feel about diseases and vaccinations and how those feelings are changing over time (12). They can also make data that public health professionals can use to help them make decisions. Sentiment analysis technologies also help doctors understand what the public wants and make changes to how popular science and public health messages are communicated (12,15).

AI chatbots offer significant advantages, particularly in addressing global disparities in medical resource distribution. Recent research indicates that within specific domains—such as standardized online consultations—AI chatbots can generate responses that quality-wise surpass those of physicians. Quantitative evaluations reveal that the average quality score of physician responses was 21% lower than that of AI chatbots. Notably, the incidence of responses deemed “unqualified” was 10.6 times higher among physicians, whereas AI chatbots produced “good or excellent” responses at a rate 3.6 times greater (16,17). However, these performance metrics are context-dependent and do not extend to complex, ambiguous, or ethically nuanced clinical scenarios, where human clinical judgment, experience, and interpersonal skills remain paramount. Consequently, AI chatbots should be conceptualized as “enhancement tools” rather than replacements for skilled practitioners. While they demonstrate superior efficacy in information retrieval and standardized communication, they cannot replicate the holistic care provided by a human physician (18).

### II. Problems with Making Short Medical Videos

The key problem in this area is that the business logic of platforms and the moral standards of medicine don't always match up. Social media algorithms usually focus on “traffic” and “time spent using the site,” which means that messages that are emotional, sensational, or provocative get more attention. The medical profession is based on the principles of “Rigor and Non-maleficence.” This change in incentives makes many creators leave their jobs to meet the needs of algorithms, which is the root of all three of the problems described below. Some doctors make false claims about surgery and use treatments or approaches that don't meet accepted standards because they care more about promoting themselves. “Information Bias” based on algorithms makes it hard for the public to understand and align with what is clinically proven. Finally, the platform's failure to enforce and monitor the professionalism of the creator has led to a lot of medical videos related to the creator, as well as misleading and marketing-oriented videos (Figure 1) (19).

**1. Proliferation of Misinformation Misleading Public Health Decisions**

There are many situations in which unqualified people, posing as qualified or licensed healthcare professionals, present pseudo-scientific claims regarding “folk remedies,” exaggeration of “treatment” effectiveness, etc., or simply promote treatments and therapies based on anecdotal evidence. Misinformation will proliferate from these posts as viral content (e.g., “high cure rate with no side effects” and “universal injection therapy”), thereby reinforcing existing myths and fears. Some of these social media posts involve misinformation regarding purported herbal medicines and “miracle cures,” leading to delays in patients receiving standard medical care and misinformation regarding proper treatment options, thereby resulting in patients abandoning formal medical care (9, 13). Conversely, a “false negative” effect may arise when patients, encouraged by rudimentary knowledge acquired from short videos, underestimate the seriousness of their symptoms and omit essential professional consultations. This phenomenon of “digital overconfidence” may result in postponed diagnoses of conditions that demand prompt intervention.

Research has shown that, on average, 37% of medical-related social media posts across various platforms contain misinformation. The average rate of misinformation based on a systematic review of the health-related domain across multiple platforms varied significantly from platform to platform. In particular, the type and amount of misinformation are influenced significantly by the architecture of each platform. For instance, platforms such as Twitter, which uses text-based content, have become a major platform for promoting the spread of vaccine conspiracy theories, with 65% of all discussions related to HPV being anti-vaccine. Conversely, YouTube, which favors visual engagement, serves as an unintentional platform for promoting “miracle cures” for non-communicable diseases, with up to 77% of video content related to non-communicable diseases containing unproven treatments. TikTok, which utilizes a short-video format, poses a unique challenge, as it allows for rapid dissemination of sensationalized self-diagnoses for mental health issues. However, the use of TikTok as a platform for misinformation has not received adequate research attention from academics or researchers in the same way as other established social media platforms.

Numerous studies have shown that social networking sites like Facebook have a lower rate of misinformation (usually less than 10%) when it comes to moderating content about health-related topics. The COVID-19 pandemic made misinformation even worse, as 30% of adults in the U.S. were exposed to conspiracy theories about the pandemic (for example, “the pandemic was man-made”) when getting information about pandemic-related topics on social media. This could affect public behavior and either encourage or discourage systemic public health issues, such as refusing to follow social isolation recommendations (20). The effectiveness of many conspiracy theories related to the COVID-19 pandemic was confirmed by meta-analysis research, which demonstrated a strong correlation between the amount of unverified health-related content that individuals were exposed to and their subsequent levels of health-related anxiety, especially among women and younger adults. In turn, patients who exhibit a pattern of cyberchondria (as defined as being excessively reliant on the Internet for health-related information) further increase patient consultations to primary care physicians due to unnecessary/redundant clinical visits, which, in turn, contradicts efforts to optimize the allocation of physician resources.

**2. Major algorithmic bias making health information inequality worse**

**Figure 3:**
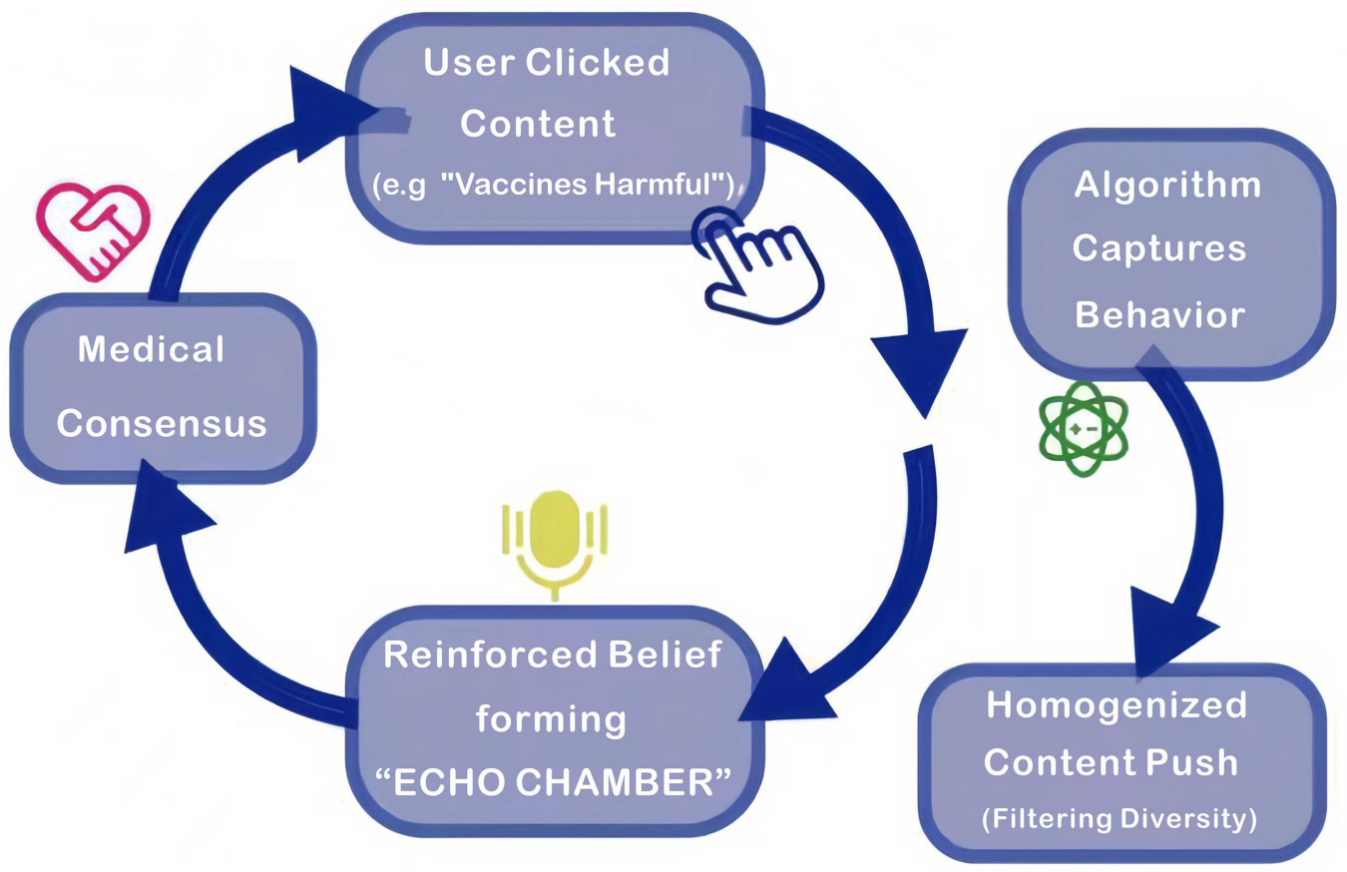
Algorithmic Echo chamber & feedback loop.

Using algorithm-driven “Feedback Loops” makes it harder for people to get health information and spreads biased information by adding more stuff. The more a user interacts with content that is based only on their existing beliefs (like the idea that “vaccines are bad”), the more content that system shows them that is like their beliefs. This “Echo Chamber” effect makes those beliefs even more different from each other, which is why cognitive polarization happens (21). (Figure 3) The elderly are especially susceptible to both impacts. Older people mostly read generic or homogenized health information, which makes them less likely to get vaccinated. Algorithmic prejudice also hurts older people who live in distant areas where they can't easily get to healthcare professionals. In these places, 61% of residents can't get health advice from tertiary hospitals because algorithms suggest viral but untrained posts. Primary care professionals also must spend 22% more time addressing false or misleading information about vaccine safety claims that their patients make. These cumulative impacts create a loop of fewer resources, which puts more strain on people. They also make people feel more alone and depressed because they don't grasp health issues accurately, which leads to bad decisions about health (22).

**3. Conflicts between business and ethics that damage the credibility of healthcare**

The health and marketing sectors have been increasingly converging. Commercialism is particularly detrimental because it undermines medical authority. “Trust Transfer,” wherein individuals—occasionally without conscious awareness—place their confidence in the endorsement signified by the “white coat” insignia on medical recommendations, will become increasingly prevalent. Doctors might take advantage of this transfer of trust to sell supplements or cosmetics that aren't backed by research while hiding conflicts of interest, which would be against the rules of their profession. “Patient Influencers” present additional ethical considerations, although their narratives are typically compelling, they are not generalizable (23). Medications that have been prescribed may be misused by patients who are influenced by laypeople. The potential to monetize “traffic” will continue to put pressure on the medical profession's ethical responsibilities. As evidence, the proliferation of commercialized misleading information in the form of medical overuse (e.g., over-diagnosis, over-treatment) is prevalent within the medical community, yet few interventions have addressed this issue. A scoping review conducted by one organization found that only 7.4% of peer-reviewed interventions have the potential to mitigate the risk of overuse, while 64% of responses by organizations/governmental organizations (e.g., FDA, Health Canada Regulations) aim to address the negative consequences of misleading claims related to unproven treatments. For example, the misleading promotion of unnecessary supplements or cosmetic procedures on social media may encourage individuals to pursue low-value care, resulting in unnecessary consumption of medical resources and potential harm, demonstrating the importance of acknowledging “overuse prevention” in the establishment of ethical governance (24).

### III. Discussion and Governance Framework

**Figure 4:**
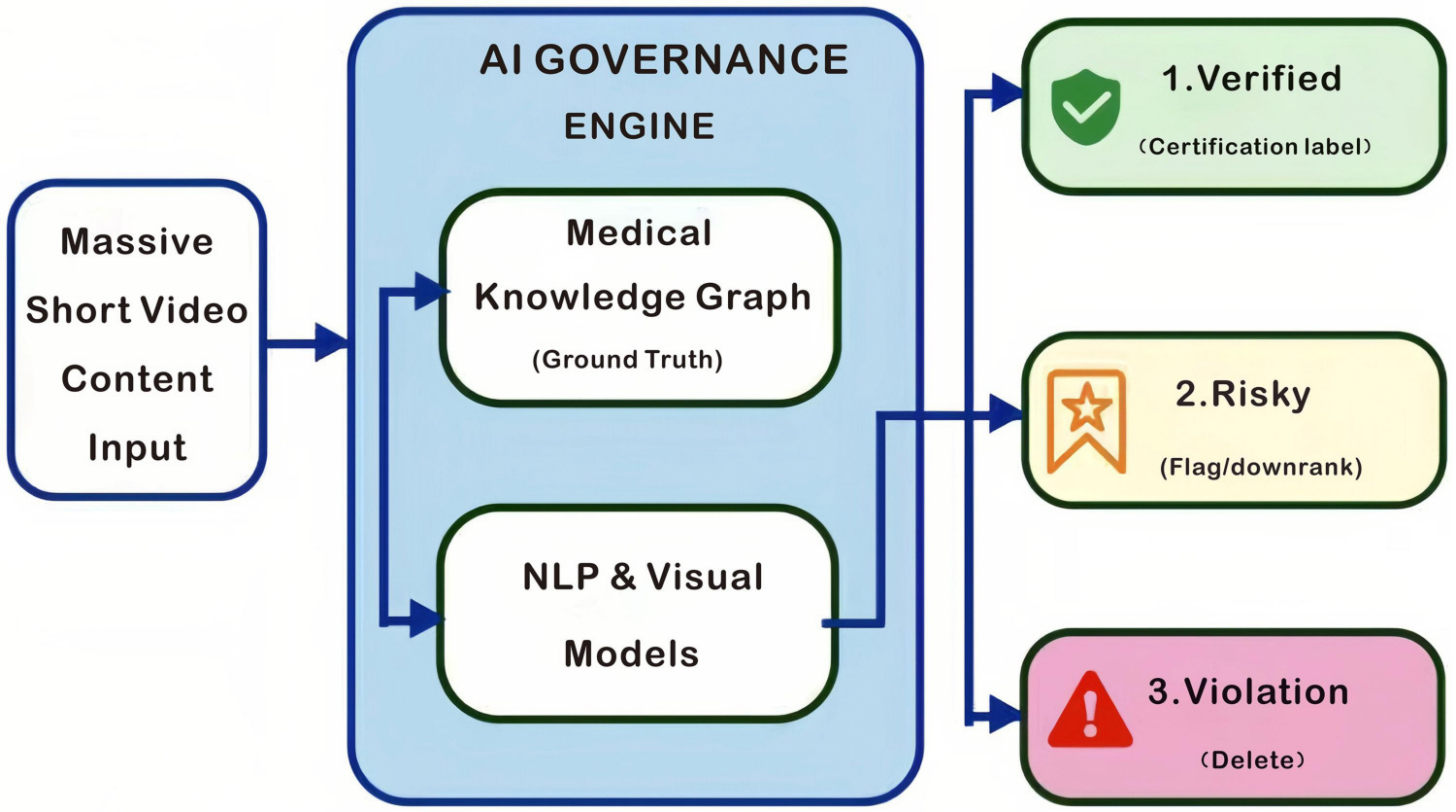
AI governance architecture flowchart.

To make the most of the benefits of short video social media sites in healthcare, a variety of stakeholders need to work together to make sure that these sites may grow safely and in an orderly manner. To do this, medical institutions need to make their presence and voice more known on digital platforms by making standardized content that follows clinically approved norms. By doing this, companies may make authoritative information on these platforms more widely available and useful. In addition to the efforts of individual institutions, public health agencies and governments should adopt a “multi-channel synergy” approach to prevent the isolation of information to only one platform. This collaborative model, which has been validated by other global partners providing health information, typically includes three components: ① Distribution of accessible, evidence-based health information via platforms that target the identified audience (e.g., Instagram, Douyin and Facebook) with the use of FAQ pages, links to peer-reviewed articles, etc. making it easier for audiences to locate, access and verify informative resources; ② Real-time updates to the respective platform's information, such as guidelines (e.g., disease prevention measures and treatment recommendations/disease treatment protocols) on the official websites, through the ability to synchronize: a record of the latest guiding principles, as well as the records of notifications that notify target audiences about revisions; ③ Proactive interaction with frontline healthcare workers (e.g., family practice, community health center workers) by encouraging them to marry the online information with the consultations/discussions that occur during in-person visits; e.g., to clarify and address patients' questions that they encountered on social media regarding what they viewed online, by creating a closed-loop online browsing-offline verification system. Also, scientists should be motivated to include their research in a formal assessment system that sets standard expectations for online behavior by being evaluated on how well they fill their online health ecosystems with high-quality health content. We need to go beyond just a passive moderation role to lower the dangers that exist without stopping innovation from happening. As indicated in Figure 4, we suggest setting up a proactive, multi-tiered AI governance strategy. In this paradigm, Natural Language Processing (NLP) algorithms that have been trained on the most recent medical literature for a certain topic would be able to find information that goes against the accepted medical consensus (for example, content about vaccine conspiracies). Also, Medical Knowledge Graphs will be used to build the “ Ground Truth,” which will be the basis for dependable verification. Finally, governance must be a shared responsibility. Medical institutions need to set standard clinical baselines and make an environment where each doctor has a stake in giving out good health information. This joint effort will help and promote public health, not just be a way to get more people to your website. This collaborative initiative will facilitate and advance public health. To facilitate effective implementation, stakeholders may consult the World Health Organization's (WHO) guidelines on digital health interventions. For example, in terms of accountability, platforms might publish regular transparency reports that specify the quantity of health content marked by AI filters and assess the precision of these measures. Similarly, medical institutions could quantitatively assess the “digital reach” of their certified content as a crucial indicator of public health influence, transitioning from passive guidelines to proactive monitoring. To establish this governance framework, the subsequent tripartite structure clearly defines the responsibilities of each group in mitigating health misinformation. (25)

To achieve this governance structure the following tripartite framework delineates each group's responsibility in alleviating health misinformation:

A Role in Institutions (The Ground Truth). For high-risk health topics like vaccines, medical institutions are the “Ground Truth” of published guideline-aligned content. They also have official ways to quickly put an end to any myths that are going around.

A Platform Role (The Filter). The platform's job is to change the way incentives work so that sensationalism doesn't happen and to create an AI-powered NLP model that will flag any information that goes against scientific medical consensus to lessen the consequences of “Echo Chambers”.

A Personal Role (The Credibility). Doctors must follow a strict code of ethics and can't use “trust transfer” to sell products like supplements. Additionally, doctors need to develop “AI literacy” as a key skill. They should act as stewards who promote social responsibility and ethical awareness when using AI technologies to make sure that the information they give to patients is accurate. Instead, they must use their professional skills to make the complicated reasons for medical treatment understandable to patients, which builds trust. This collaboration between three groups will make it feasible to comprehend the full range and complexity of health misinformation.

## Data Availability

The original contributions presented in the study are included in the article/Supplementary Material, further inquiries can be directed to the corresponding author.
